# Potential Diagnostic and Prognostic Biomarkers of Circular RNAs for Lung Cancer in China

**DOI:** 10.1155/2019/8023541

**Published:** 2019-08-25

**Authors:** Chengdi Wang, Yuting Jiang, Qian Lei, Yangping Wu, Jun Shao, Dan Pu, Weimin Li

**Affiliations:** ^1^Department of Respiratory and Critical Care Medicine, West China Medical School/West China Hospital, Sichuan University, Chengdu, China; ^2^West China Medical School, Sichuan University, Chengdu, China; ^3^Department of Targeted Tracer Research and Development Laboratory, West China Hospital, Sichuan University, Chengdu, China; ^4^Department of Clinical Research Management, West China Hospital, Sichuan University, Chengdu, China; ^5^Clinic Skill Center, West China Hospital, Sichuan University, Chengdu, China

## Abstract

Emerging evidence demonstrated that circular RNAs (circRNAs) were dysregulated in lung cancer, indicating that circRNAs might serve as novel diagnostic and prognostic biomarkers for lung cancer. However, the clinical value of circRNAs on lung cancer remains unclear. This study aimed to evaluate the efficiency of circRNAs in the diagnosis and prognosis for lung cancer in China. 2122 Chinese individuals were enrolled in this investigation for assessment of diagnostic value and examination of prognostic analysis. In the diagnostic analysis, the pooled sensitivity, specificity, PLR, NLR, DOR, and AUC of the sROC curve with their 95% CIs were 0.80 (95%CI: 0.74-0.84), 0.80 (95%CI: 0.73-0.86), 3.97 (95%CI: 2.80-5.62) and 0.26 (95%CI: 0.19-0.34), 15.51 (95%CI: 8.76-24.47), and 0.85 (95%CI: 0.82-0.88), respectively. As for the prognostic power of circRNAs, lung cancer patients with higher expression levels of circRNAs tend to possess lower overall survival with the overall pooled HR (1.70, 95%CI: 1.26-2.29). Furthermore, in stratified analysis, upregulated and downregulated circRNAs were manifested to exert significant effects on prognosis with HR values of 2.17 (95%CI: 1.74-2.72) and 0.52 (95%CI: 0.34-0.80). This study validates that circRNAs are promising diagnostic and predictive biomarkers for lung cancer patients in China.

## 1. Introduction

Lung cancer is the most common of new cancer cases accounting for 11.6% of the new diagnosed cases and ranks as the leading cause of cancer death sharing 18.4% of the overall cancer mortality [1]. By sex, in males, lung cancer is the top one cancer type, responsible for newly diagnosed cancer patients and death, whereas lung cancer is the second death cause inferior to breast cancer and as for incidence rate comes in third behind breast cancer and colorectum cancer among females [1]. Obviously, lung cancer has been a major public health problem worldwide especially in China [2].

Despite the considerable efforts exerted on dealing with cancer, there are still clinical challenges in cancer management, mainly ascribed to low early metaphase diagnosis rate with cancer, ineffective treatment, and uncertainty about clinical outcomes. Early diagnosis can make a huge difference to lung cancer patients, for providing the best opportunity for medical support [3]. If diagnosed at early stage, lung cancer patients with mild symptoms may be protected from developing severe, late-stage, and advanced cancer types, which will tend to require more intricate and expensive treatment with poorer curative effects. Owing to the reasons mentioned above, traditional treatments are not satisfactory [4]. Immunotherapy, stem cells, and genomic medicine are emerging as novel attractive candidate strategies against cancer with striking treatment effect. But more importantly, there are yet a substantial number of obstacles to overcome, such as second developed drug resistance, prior to entering the clinic and being widely employed. Thus, effective methods or biomarkers are in extremely urgent need for early diagnosis and prognosis of lung cancer, so as to monitor the progress of cancer and adjust treatment plan timely.

Circular RNAs (circRNAs) are emerging as a promising biomarker for cancers [5]. CircRNAs distinctly feature covalently closed continuous loop structures without 3' ends and 5' ends, while, in well-established linear RNAs, another important member of the family of endogenous noncoding RNAs, 3' ends and 5' ends exist, limit the direction of synthesis of nucleic acids in vivo to 5'-to-3', and contribute to linear RNAs' sensitivity to nuclease [6, 7]. At first, circRNAs were noted as abnormal byproducts of back-splicing of pre-mRNA transcription because pretty low expression levels of circRNAs were observed [8]. However, with the burgeoning development and incremental application of novel technologies, especially the high-throughput RNA sequencing, altered circRNAs are confirmed to be ubiquitously expressed [9, 10].

More importantly, the distinct molecular structure grants circRNAs multiple nature, including stability, specificity, and conservation across mammals [9, 11]. Compared with linear RNAs, circRNAs can avoid exonucleolytic degradation by RNase; thereby, they tend to possess longer half-time and then are able to stay more stable in vivo over an extended period [12], which partly conduce to plentiful expression in internal environment. Moreover, circRNAs are deemed to be dispersed in a cell/tissue-dependent manner and their expression levels vary, which is consistent with specific developmental stages [12–14], which are related to their extensive biological functions, involved in cell proliferation, differentiation, migration, invasion, and apoptosis [15–21]. At the same time, increasing evidence, focused on the correlation between circRNAs and clinical characteristics of cancer sufferers, revealed that circRNAs might act as effective diagnostic biomarkers and forecast clinical outcomes of cancer patients [22]. Zhao et al. [23] screened 357 differentially expressed circRNAs by high-throughput sequencing in early lung adenocarcinoma. They further investigated 5 circRNAs by bioinformatic analysis and reported that these circRNAs might function as diagnostic markers in cancer. What is more, circFOXO3, a putative tumor suppressor, was significantly downregulated in lung cancer and breast cancer [24, 25]. Zhang et al. reported that circFOXO3 served as a novel biomarker for early diagnosis with AUC of 0.782 in lung cancer and in vitro investigations implied inversely correlation with migration and invasion of nonsmall cell lung cancer through sponging miR-155 and releasing FOXO3 gene [24]. Besides, CiRS-7 (circular RNA sponge for miR-7), also termed CDR1as (cerebellar degeneration-related protein 1 transcript), harbors more than 70 conventional miR-7 binding sites and directly suppresses activity of miR-7 [26]. Apart from being a well-known tumor suppressor, miR-7 is also reported to show the opposite influence effect in lung cancer [26–28], colorectal cancer [29, 30], and hepatocellular carcinoma [31]. Upregulated expression levels of CDR1as with concomitant underexpressed mir-7 were proved to closely relate to high TNM stage, lymph nodes metastasis, and short survival time [32]. On the contrary, Chou and his colleagues identified overexpression of miR-7 with carcinogenesis and poor prognosis of lung cancer. Analogously, in the context of inhibition of miR-7, there was reduced proliferation of lung cancer cell lines [28].

Thus, there are disagreements and inconformity among the results of diverse studies concentrated on the diagnostic ability and prognostic value of circRNAs. Here, we performed a comprehensive and quantitative study to summarize the diagnostic and prognostic utility of circRNAs in human lung cancer specifically, tried to clarify and address the discrepancy among researches, and expected to furnish guideline to clinical management of lung cancer.

## 2. Materials and Methods

### 2.1. Search Strategy and Study Selection

A comprehensive search was conducted to identify potential articles published in English up to December, 2018, from PubMed, PMC, EMBASE, Web of Science, Cochrane Library, China National Knowledge Infrastructure Database (CNKI), Wanfang Database, and China Biological Medicine Database (CBM). The search terms employed for literature retrieval were (circRNA OR circular RNA) AND (lung cancer OR lung carcinoma OR pulmonary carcinoma OR pulmonary cancer OR lung squamous cell carcinoma OR non-small-cell lung cancer OR small cell lung cancer). Reference lists of relevant papers were obtained manually to identify potential eligibility.

Two investigators (Y. T. Jiang and J. Shao) independently perused the full texts of potentially eligible studies based on their titles and abstracts. Any disagreement was resolved until a consensus was reached with a third researcher (C. D. Wang).

Publications included in this meta-analysis fulfilled the following criteria: (1) case‐control study or cohort study including both case and control groups; (2) patients with a pathological diagnosis of lung cancer; (3) studies estimating performance of circRNAs for the diagnosis or predicting the outcome of lung cancer patients; (4) the sensitivity and specificity data or HRs with 95% CI (or the possibility of deriving such statistics from the manuscript) that were available. And exclusion criteria included (1) studies not relevant to circRNA or lung cancer; (2) key information or usable data that were missing; (3) duplicated publications; (4) reviews, letters, case reports, summaries of conference, and so on. If articles were published based on overlapping data by the same author, only the most complete study was included.

### 2.2. Data Extraction and Quality Assessment

Data are collected according to different study types.

(I) In studies using circRNAs as diagnostic marker for lung cancer, following data were enrolled: the first author, publication year, country and ethnicity, cancer type, specimen source, sample size, cut-off value, area under the curve (AUC), data for 2 × 2 contingency table (sensitivity and specificity), and detection method.

(II) In these articles assessing prognostic significance of circRNAs in lung cancer, we extracted following information: the first author, publication year, country and ethnicity, cancer type, specimen source, sample size, cut-off value, follow-up time (month), treatment, and HR values of evaluated circRNAs for overall survival (OS) analysis as well as their 95% CI and P value.

Two researchers reviewed and evaluated the quality of studies enrolled in prognostic analysis based on the guideline of The Quality Assessment of Diagnostic Accuracy Studies 2 (QUADAS-2) checklist. QUADAS-2 consists of 14 items, and there is an answer of “Yes,” “unclear,” and “No” for each item for which only “Yes” scores one point. The Newcastle-Ottawa Quality Assessment Scale (NOS) was adopted to systematically assess articles included in the prognostic meta-analysis. Specifically, the cut-off point is defined as 6. Higher scores represent better reporting quality.

### 2.3. Statistical Analysis

All statistical analyses were performed with STATA version 15.0 (STATA Corporation LLC, Texas, USA) and Review Manager 5.3 (Cochrane Collaboration, London, UK). Pooled sensitivity, specificity, positive likelihood ratio (PLR), negative likelihood ratio (NLR), diagnostic odds ratios (DOR) and their 95% confidence interval (CI), summary receiver operator characteristic (SROC) curve, and area under the curve (AUC) were calculated to estimate the ability of circRNA to distinguish lung cancer patients from healthy people. As for survival rates, all provided HRs as well as 95%CI were obtained to study the overall performance of the prognostic test. P<0.05 (two-sided) was considered as a statistically significant difference. Heterogeneity across studios was tested by Cochran's Q test and Higgins's* I*^2^ statistics. A random-effect model was utilized when P < 0.10 and* I*^2^ > 50%, indicating the presence of heterogeneity. Otherwise, the fixed-effect model was carried out. Finally, publication bias was described by Egger's bias indicator test.

## 3. Result

### 3.1. Study Selection

**Figure 1:**
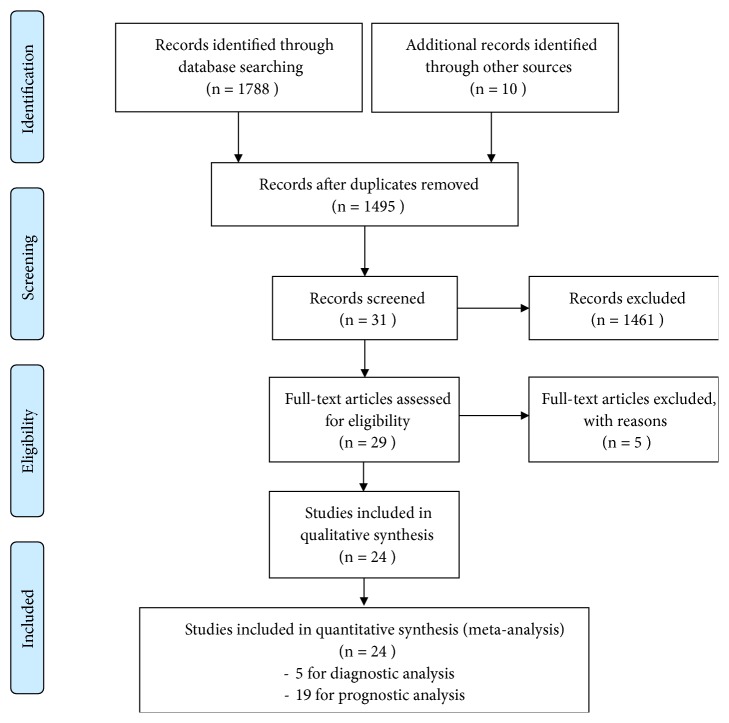
Flow diagram of the study selection process. Totally, 1798 were identified through database or by searching manually. And 24 articles were enrolled in the final analysis including 5 diagnostic studies and 19 prognostic studies.

A total of 1798 potentially relevant articles were initially identified. After abstract and full article review, 24 published articles were enrolled for the final analysis. Among them, 5 articles investigated diagnostic value of circRNAs in lung cancer [24, 33–36], and 19 studies examined prognostic information related to overall survival [32, 37–54]. The period of the eligible studies ranged from 2017 to 2018 with a total of 2122 individuals. The process of selection is shown (Figure 1).

### 3.2. Study Characteristics and Quality Assessment

The characteristics of 24 eligible studies are summarized in Tables 1 and 2. Among them, 20 circRNAs were upregulated in lung cancer patients while 4 types were downregulated (circFOXO3, Hsa_circ_0001649, Hsa_circ_0046264, and Hsa_circ_100395). All of the sources of sample were tissue. The sample size ranged from 43 to 184. And the overall size in diagnostic meta-analysis was 578 and in the patients involved in prognostic analysis it was 1544. The cut-off values were not consistent in included studies. Additionally, we evaluated the quality of publications concerning diagnosis by QUADAS-2 ([Supplementary-material supplementary-material-1]) and detailed information was shown in Table 1, demonstrating reliable foundation of this study. The quality of prognostic articles was assessed by NOS, and quality scores more than 6 were recognized as high quality in Table 2. The median of involved studies was 8, which indicated that the inclusive articles were of good quality.

### 3.3. Diagnostic Accuracy Analysis

**Figure 2:**
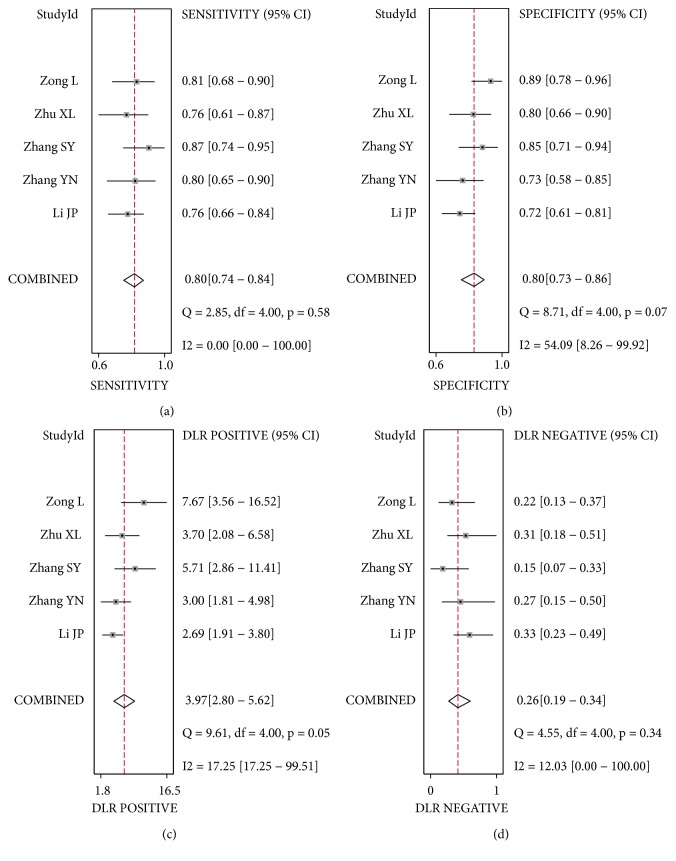
Forest plots of diagnostic accuracy index of circRNAs in lung cancer. (a) Sensitivity of circRNAs in diagnosis of lung cancer. (b) Specificity of circRNAs in diagnosis of lung cancer. (c) Positive likelihood ratio of circRNAs in diagnosis of lung cancer. (d) Negative likelihood ratio of circRNAs in diagnosis of lung cancer.

**Figure 3:**
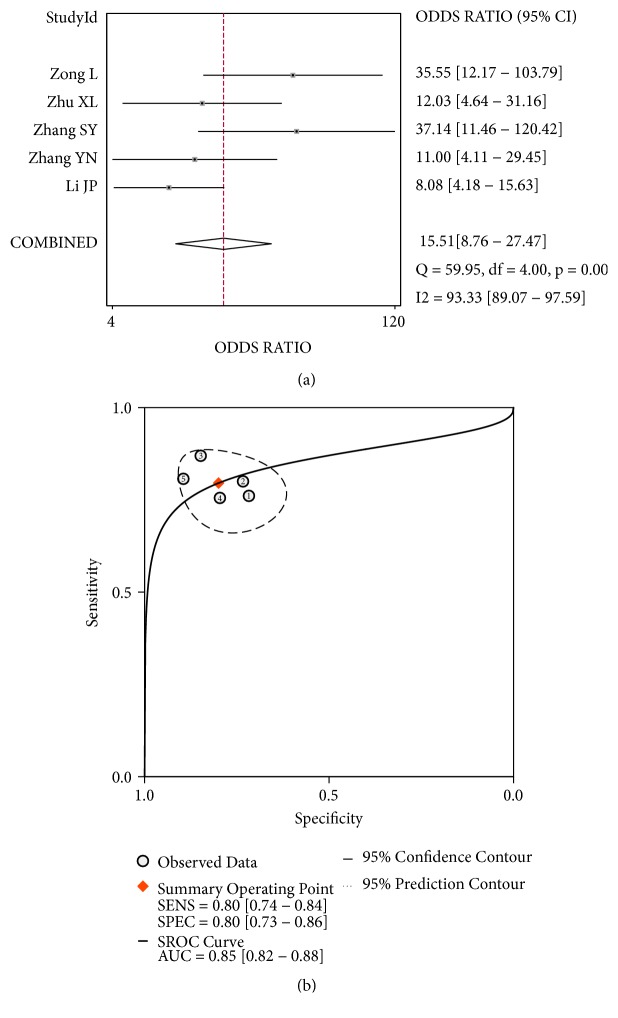
Overall performance of circRNAs in diagnosis of lung cancer. (a) Diagnostic odds ratio of circRNAs in diagnosis of lung cancer. (b) Summary receiver operator characteristic curve of circRNAs in diagnosis of lung cancer.

**Figure 4:**
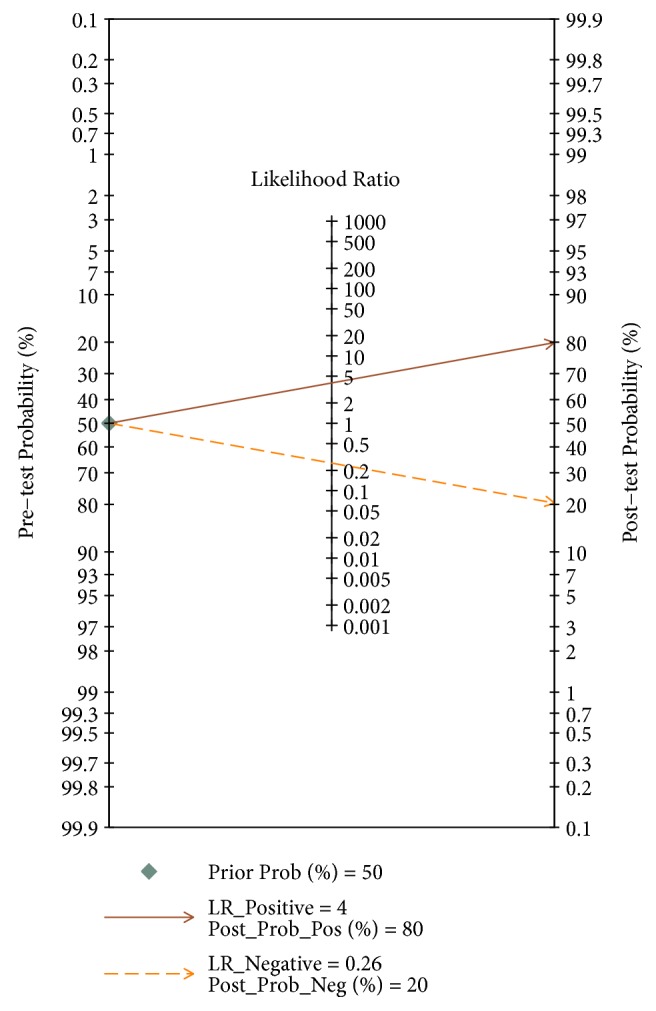
Fagan's nomogram estimating the overall value of circRNAs in cancer detection.

The pooled sensitivity (Figure 2(a)), specificity (Figure 2(b)), PLR (Figure 2(c)), and NLR (Figure 2(d)) with their 95% CIs were 0.80 (95%: 0.74-0.84), 0.80 (95%: 0.73-0.86), 3.97 (95%: 2.80-5.62), and 0.26 (0.19-0.34), respectively. The pooled DOR (Figure 3(a), 15.51, 95%CI: 8.76-27.47) and AUC (Figure 3(b), 0.85, 95%CI: 0.82-0.88) of the SROC curve were utilized to assess the overall diagnostic performance. Nomogram of Fagan was utilized and the results were demonstrated (Figure 4). The diagnostic performance was summarized in Table 3.

### 3.4. Prognostic Value of CircRNA Expression for Cancer Survival

**Figure 5:**
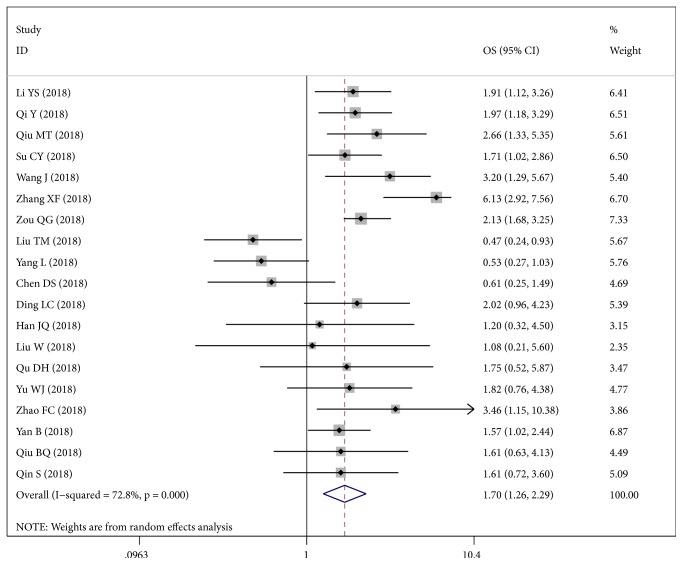
Forest plots of the overall prognostic performance of circRNAs.

**Figure 6:**
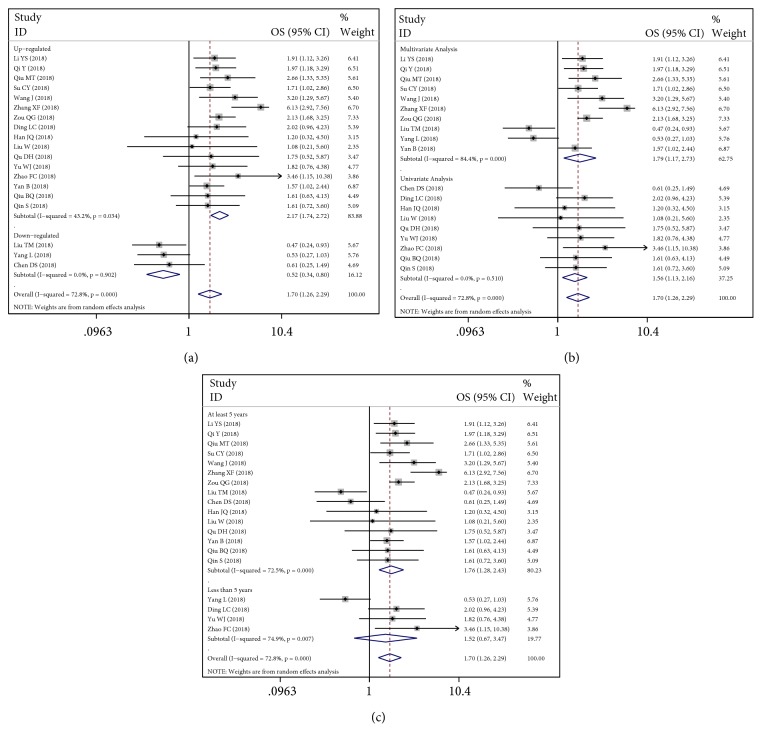
Meta-analysis of subtotal HRs based on (a) upregulated and downregulated circRNAs, (b) analysis methods, and (c) follow-up time.

Totally, 10 studies provided reported overall survival data and 9 articles concerning Kaplan-Meier curves were calculated to obtain HRs and their 95%CIs. The pooled HR is 1.70 (95%:1.26-2.29) with significant heterogeneity (*I*^2^:72.8%). The overall performance of circRNA as a prognostic biomarker was illustrated (Figure 5). Thus, subgroup analysis was conducted in Table 4. First, upregulated and downregulated circRNAs were analyzed to obtain their HRs values (Figure 6(a)). The recalculated HRs are 2.17 (95%CI: 1.74-2.72) and 0.52 (95%CI: 0.34-0.80) with low heterogeneity (*I*^2^: 43.2% and 0.00%, respectively). And there was obviously statistical significance either in multivariate analysis or in univariate analysis (p: 0.007, Figure 6(b)). There was a significant association between more than 5-year period time and survival, indicating 5-year follow-up is necessary (Figure 6(c)).

### 3.5. Publication Bias and Sensitivity Analyses

**Figure 7:**
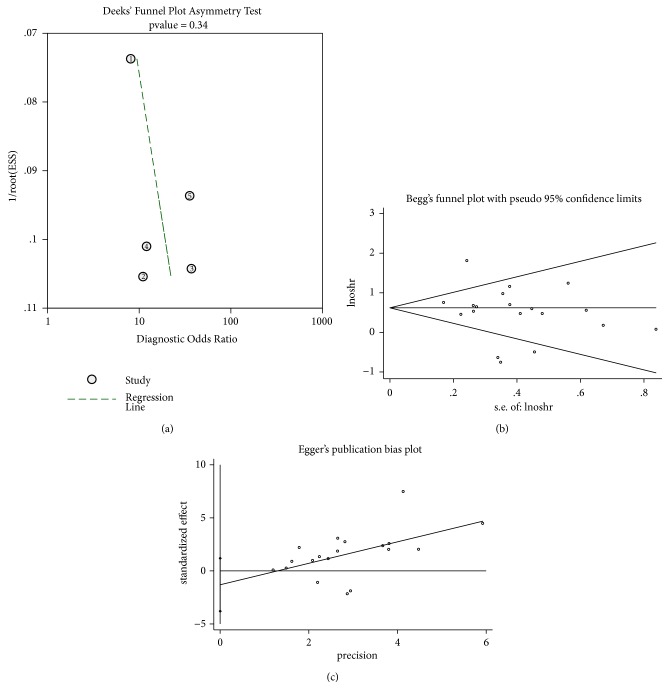
Funnel plots of publication bias. (a) Deeks' funnel plot asymmetry test for diagnostic studies. (b) Begg's funnel plot for prognostic tests. (c) Egger's funnel plot for prognostic tests.

**Figure 8:**
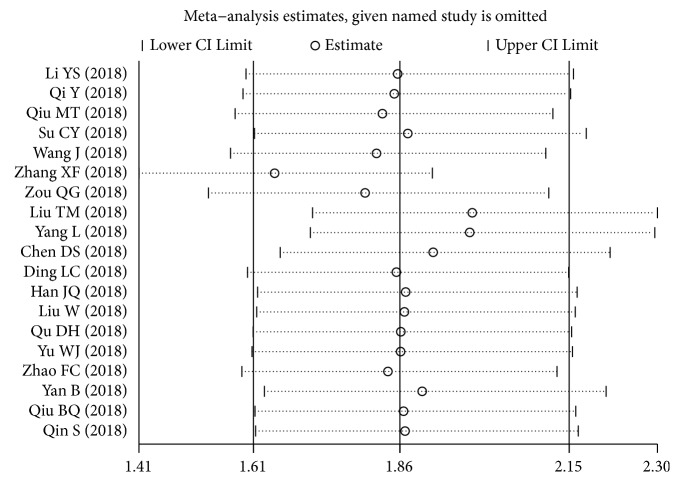
Sensitivity analyses for prognostic analysis.

The publication bias of diagnostic studies was checked by Deeks' test (P=0.34, Figure 7(a)), indicating no potential bias. As for prognostic articles, the p values of Begg's and Egger's test were 0.484 and 0.339, respectively (Figures 7(b) and 7(c)). Thus, there were no publication biases in the studies enrolled in the current study. Then, through successively omitting each prognostic individual study, the consequence was not significantly influenced, indicating that the result of this study was robust (Figure 8).

## 4. Discussion

As a member of noncoding cancer genomes, circRNAs gradually attract worldwide attention because accumulating evidence revealed various functions of circRNAs with an emphasis on their association with cancer. Due to being insensitive to RNase, circRNAs tend to keep stable and specifically exist in the plasma of lung cancer patients like F-circEA, which implies that circRNAs may be employed as noninvasive diagnostic biomarkers [12, 55]. Ubiquitously existing in body, altered expression levels of circRNAs are disease specific or often predict prognosis [56]. Therefore, circRNAs may be used as biomarkers so as to facilitate early diagnosis and improvement on prognosis of lung carcinoma. Previous reviews focused on correlation between circRNAs and multiple cancers, but none of them investigated on lung cancer specifically. We summarized recent studies of circRNAs in lung cancer, highlighting circRNAs as diagnostic and prognostic tools. Thus, this study is the first meta-analysis to direct at and summarize the potential diagnostic and prognostic roles of circRNAs for human lung cancer specifically, hoping to contribute to a better and deeper understanding of the complex relationship between the various expression levels of circRNAs and lung cancer.

### 4.1. CircRNAs Are Diagnostic Biomarkers for Lung Cancer

We retrieved 5 published articles pertaining to the expression levels of different circRNAs in human lung cancer, including 1 downregulated circRNA [24] and 4 upregulated circRNAs [33–36]. Furthermore, in the selection process for eligible articles, results of studies were considered acceptable only based on the expression of circRNAs in tissue, while consequences of studies based on the expression levels of circRNAs in serum, plasma, or peripheral blood samples were not taken into account.

CircFOXO3 was observed with decreased expression in nonsmall cell lung cancer and related to clinical diagnosis with AUC of 0.782 [24]. A study by Lu and his colleagues confirmed that circFOXO3 is significantly downregulated in breast tumor as well [25]. But compared with that in healthy controls, the expression of hsa_circ_0013958 significantly increased in stage I/II lung adenocarcinoma patients [33]. Similarly, the expression levels of hsa_circ_0079530, hsa_circ_0014130, hsa_circ_102231, and hsa_circ_0000729 were upregulated in lung cancer with good sensitivity and specificity [34–36, 57].

On account of the inconsistent or opposite results of these included studies, several statistical tools were employed to assess the overall diagnostic performance of circRNAs in lung cancer. The sensitivity and specificity were performed to measure the diagnostic value and the pooled sensitivity and specificity were 0.80 (95%: 0.74-0.84) and 0.80 (95%: 0.73-0.86), indicating moderate strength to detect lung cancer. In addition, DOR is a single indicator of overall effectiveness of a diagnostic test and when it is greater than one, the test is discriminating correctly [58]. The pooled DOR herein was 15.51 with corresponding 95% confidence interval from 8.76 to 27.47 and it suggested that circRNAs involved in our study possessed satisfactory ability of diagnosis. Another recommended implement is the AUC of the summary receiver operating characteristic curve (SROC), representing the value of a diagnostic experiment. It is generally recognized that the AUC of SROC with a value more than 0.93 is good and a value ranging from 0.75 to 0.92 is receivable [59, 60].

In the current study, the value of AUC was 0.85 (95%CI: 0.82-0.88). Given the results discussed hereinabove, circRNAs are capable for early detection of lung cancer. Since current conventional serum biomarkers such as carcinoembryonic antigen, cytokeratin 19 fragments 21-1, and neuron-specific enolase are unsatisfactory in both sensitivity and specificity of early detection of lung cancer, circRNAs, with a pooled sensitivity and specificity of 0.8 and 0.8, respectively, are relatively hopeful indicators so as to contribute positively to the improvement in the early diagnosis with lung cancer.

Obvious heterogeneity of this diagnostic analysis was assessed; however, we were not able to perform stratified analysis to find out the source of heterogeneity due to lack of sufficient data about some crucial variates concerning design schema, country, ethnicity, age, circRNAs type, controls type, and so on.

### 4.2. CircRNAs Are Prognostic Biomarkers for Lung Cancer

In the present study, 17 types of circRNAs from 19 studies were identified for prognostic value in lung cancer. All the included studies concentrated on the relationship between aberrant expression of circRNAs and overall survival of lung cancer patients and none of them dealt with other survival indexes like progress free survival. It was mentioned that all patients involved in the study did not receive radiotherapy or chemotherapy before surgery when samples were acquired. Among 19 studies, 3 studies of circRNAs were downregulated, including hsa_circ_0001649, hsa_circ_0046264, and hsa_circ_100395, while the expression levels of the remaining 16 studies of circRNAs were in the opposite.

On the whole, our results revealed that the upregulated circRNAs were related to a worse overall survival for lung cancer patients as the pooled HR was 1.70 with 95% CI from 1.26 to 2.29. Because there was an evident heterogeneity, subgroup analyses were employed to explore the source. According to the biological function of circRNAs, the upregulated biomarker group showed a lower OS with the pooled HR of 2.17 (p<0.001) and pretty low heterogeneity (I^2^: 43.2%), whereas the downregulated group were investigated to have a significantly positive correlation with a stronger prognosis (HR: 0.52, p: 0.002) and improved heterogeneity. Besides, the diversity of analysis methods used in the enrolled research may have an impact on the final results. Multivariate analysis takes into account of all statistical outcome variables at the same time while univariate analysis is conducted with a single factor, considered as the simplest form of quantitative analysis. Generally, multivariate analysis tends to demonstrate higher statistical accuracy than univariate analysis. Similarly, longer follow-up time will be more useful for further evaluation of prognostic values in complex diseases, especially in lung cancer. However, analysis methods in original articles for HR and corresponding 95%CI and the length of the time for following up were discovered with nonsignificant association with lung cancer patients' overall survival, meaning that the heterogeneity was not amended after these subset analyses. So we concluded that merging the types of circRNAs with distinctly different biological roles might explain the main source of the heterogeneity.

The overexpression of circPRKCI, circCDR1as, circBANP, and circFADS2 was correlated with unfavorable prognosis in lung cancer. However, circP4HB, circSHPRH, and hsa_circ_100395 were decreased in lung cancer tissues and low expression predicted poor prognosis. Accumulating evidence revealed that the circPRKCI, circCDR1as, circBANP, and circFADS2 functioned as an oncogenic role in lung cancer, whereas the circP4HB, circSHPRH, and hsa_circ_100395 act as tumor suppressor of lung cancer.

In mechanism, aberrant circPRKCI inhibits the cellular proliferation, distant metastasis, and cell invasion in lung cancer by modulating the expression of markers of epithelial-to-mesenchymal transition, sponging miR-545 and miR-589, and relieving the inhibition of the protumor genic transcription factor E2F7 [44]. Besides, circCDR1as, one of the most frequently studied circRNAs, targets miR-7 in a manner dependent on NF-kB regulatory signaling, upregulates proliferation levels of EGFR, CCNE1, and PIK3CD, and thus induced superior proliferative, migratory, and invasive capabilities of lung cancer cells [12, 32, 49, 61]. Upregulation of ciRS-7 was also identified in colorectal carcinoma and hepatocellular carcinoma with shorter patient survival time than patients with low ciRS-7 expression [30, 31]. What is more, remarkably unregulated circBANP promoted lung cancer cells proliferation and invasion by abrogating the antitumor effects of miR-503/LARP1[39]. Similarly, high expression of circBANP was observed in colorectal carcinoma [62]. Moreover, Circ-FADS2-mediated miR-498 signaling pathway contributes to lung cancer growth and viabilities, and the patients with low expression level of circFADS2 were considered to have favorable clinical outcomes [50]. On the contrary, in basal cell carcinoma and cutaneous squamous cell carcinoma, Sand et al. demonstrated that the two most downregulated circRNAs were derived from the FADS2 gene and they promoted tumor cell proliferation and tumorigenesis [63, 64].

In terms of tumor suppressors, circITCH was another well-established molecular biomarker. Expression of circITCH was decreased in colorectal carcinoma [65], lung cancer [66], and esophageal squamous cell carcinoma [67]. CircITCH prevented Wnt/*β*-Catenin pathway from activation and exerted inhibition effects on the progression of lung cancer through sponging the miR-7 and miR-214. Hsa_circ_100395 was found to serve as a sponge for TCF 21 in lung cancer and expression level of hsa_circ_100395 was inversely associated with lymph node metastasis and Tumor-Node-Metastasis stage [37]. CircSHPRH was confirmed as the sponge miR-331-3p and miR-338-5p and thus inhibiting lung cancer cell growth and metastasis [41]. A similar result was observed in Qin's study that the expression of circSHPRH was significantly reduced in hepatocellular carcinoma [17]. As for circP4HB, it promoted apoptosis, yet it arrested cell-cycle progression, restrained proliferation, and reduced cell invasion and migration through upregulating BRCA2 via targeting miR-1245 [47].

Despite the fact that great efforts were paid to fulfill this systematic and comprehensive analysis based on credible quality of included studies, there were still some deficiencies in our study. First, the majority of sample size of subjects is small [68, 69]. The detection of circRNAs mainly relies on the high throughput sequencing, which is relatively more expensive than traditional detection technology; as a result, the wide clinic application of circRNAs is limited by the costly test method. Equally, the high throughput sequencing, emerging in the last decade, leads to the fact that the researches concentrated on the diagnostic or prognostic roles of circRNA are confined to recent years, most of which are in the year of 2018. Second, the county studied was restricted to China. It was noteworthy that, as a developing country, incidence of various cancer types that occurred in China was different from developed country [70]. Although the morbidity of lung cancer in China accounts for approximately one-third of global new diagnosed cases [1], the imperfection of populations researched narrowed the ranges of applicability in terms of diverse genetic backgrounds and geographic disparity. Third, the heterogeneity of overall diagnostic accuracy and predictive significance was evident, and the potential sources of heterogeneity were not duly clarified by satisfying subgroup analysis owing to insufficient data, which were vital to describe effectiveness of circRNAs in a quantitative manner. Fourth, the samples were extracted merely from lung tissue. CircRNAs that are characterized with closed loop structure, free of poly(A) tails, and the feature which confers them advantageous properties that altered expression of circRNAs are confirmed to be ubiquitous and stable in various human organs and developmental stages. Hence, it was inappropriate to discard records with other sources of samples like peripheral blood mononuclear cell [56] in the literature selection process. Furthermore, given that cancer is something pervasive and stubborn, with sophisticated and underlying mechanisms, it is suggested that combination of several biomarkers might exert better diagnostic accuracy or higher prognostic value of lung cancer than a single biomarker. Besides, more and more tumor markers are implied to appear in a tissue specific manner and serve to distinguish the organogenesis of cancer cells. Thus, further feasible researches are required to spotlight the clinical diagnostic power of detection of multitumor markers from tissue, serum, plasma, and so on, to seek for novel practical methods, to facilitate early diagnosis, and to improve clinical outcomes.

## 5. Conclusions

In summary, this study validates that the altered expressions of circRNAs can be monitored and applied as emerging diagnostic biomarkers with moderate sensitivity and specificity and have satisfactory value in forecasting clinical outcomes of lung cancer patients in China. Nevertheless, well-designed and large-scale researches of multinational clinical trials are further required to verify the results.

## Figures and Tables

**Table 1:** Characteristics and quality assessment of studies included in diagnosis meta-analysis.

First Author	Published Year	Country	Ethnicity	Cancer type	CircRNA type	Name of the host gene	Expression	Specimen source	No. of patients	No. of control	Cutoff value	AUC	TP	FP	FN	TN	Sensitivity	Specificity	Detection method	QUADAS
Li JP	2018	China	Asian	NSCLC	Hsa_circ_0079530	ACP6	U	Tissue	92	92	1.9	0.756	70	26	22	66	76.29%	72.1%	qRT-PCR	5
Zhang YN	2018	China	Asian	NSCLC	CircRNA-FOXO3	FOXO3	D	Tissue	45	45	NA	0.782	36	12	9	33	80.0%	73.3%	qRT-PCR	5
Zhang SY	2018	China	Asian	NSCLC	Hsa_circ_0014130	/	U	Tissue	46	46	0.573	0.878	40	7	6	39	87%	84.8%	qRT-PCR	4
Zhu XL	2017	China	Asian	LAC	Hsa_circ_0013958	/	U	Tissue	49	49	0.00101	0.815	37	10	12	39	75.5%	79.6%	qRT-PCR	5
Zong L	2018	China	Asian	LAC	Hsa_circ_102231	/	U	Tissue	57	57	NA	0.897	46	6	11	51	81.2%	88.7%	qRT-PCR	5

NSCLC, nonsmall cell lung cancer; LAC, lung adenocarcinoma; U, upregulated expression; D, downregulated expression; AUC, area under the curve; TP, true positive; FP, false positive; FN, false negative; TN, true negative; qRT-PCR, real-time polymerase chain reaction; QUADAS, Quality Assessment of Diagnostic Accuracy studies.

**Table 2:** Clinical characteristics and quality evaluation of articles enrolled in the prognosis analysis.

First Author	Published Year	Country	Ethnicity	Cancer type	CircRNA type	Name of the host gene	Expression	Specimen source	No. of patients	No. of control	Cutoff value	Follow-up time (month)	Treatment	OS (HR)	OS (LL)	OS (UL)	NOS Score
Li YS	2018	China	Asian	NSCLC	hsa_circ_0016760	SNAP47	U	Tissue	45	38	mean	60	Surgery	1.91	1.119	3.259	8
Qi Y	2018	China	Asian	NSCLC	hsa_circ_0007534	DDX42	U	Tissue	56	42	mean	60	Surgery	1.969	1.177	3.293	8
Qiu MT	2018	China	Asian	LAC	circ-PRKCI	PRKCI	U	Tissue	55	34	mean	80	Surgery	2.664	1.327	5.347	8
Qin S	2018	China	Asian	NSCLC	circ-PVT1	PVT1	U	Tissue	43	47	median	60	Surgery	1.61	0.72	3.60	8
Qiu BQ	2018	China	Asian	NSCLC	circ-FGFR3	FGFR3	U	Tissue	34	29	mean	80	Surgery	1.61	0.63	4.13	9
Su CY	2018	China	Asian	NSCLC	ciRS-7	CDR1as	U	Tissue	77	51	mean	60	Surgery	1.705	1.02	2.86	9
Wang J	2018	China	Asian	NSCLC	hsa_circ_0067934	PRKCI	U	Tissue	79	80	median	60	Surgery	3.198	1.293	5.673	9
Zhang XF	2018	China	Asian	NSCLC	ciRS-7	CDR1as	U	Tissue	41	19	median	70	Surgery	6.132	2.923	7.556	8
Zou QG	2018	China	Asian	NSCLC	hsa_circ_0067934	PRKCI	U	Tissue	41	38	median	60	Surgery	2.133	1.677	3.251	8
Ding LC	2018	China	Asian	NSCLC	hsa_circ_001569	/	U	Tissue	29	27	mean	50	Surgery	2.02	0.963	4.233	9
Han JQ	2018	China	Asian	LC	circ-BANP	BANP	U	Tissue	28	31	median	60	Surgery	1.196	0.323	4.496	8
Liu W	2018	China	Asian	LC	hsa_circ_103809	/	U	Tissue	22	22	mean	80	Surgery	1.08	0.21	5.60	6
Qu DH	2018	China	Asian	NSCLC	hsa_circ_0020123	PDZD8	U	Tissue	40	40	median	60	Surgery	1.747	0.52	5.867	8
Yan B	2018	China	Asian	NSCLC	ciRS-7	CDR1as	U	Tissue	66	66	median	90	Surgery	1.575	1.016	2.440	8
Yu WJ	2018	China	Asian	NSCLC	hsa_circ_0003998	ARFGEF2	U	Tissue	32	28	mean	40	Surgery	1.82	0.76	4.38	7
Zhao FC	2018	China	Asian	LC	circ-FADS2	FADS2	U	Tissue	22	21	median	50	Surgery	3.46	1.15	10.38	7
Liu TM	2018	China	Asian	NSCLC	hsa_circ_0001649	SHPRH	D	Tissue	22	31	mean	60	Surgery	0.471	0.238	0.934	8
Yang L	2018	China	Asian	LC	hsa_circ_0046264	P4HB	D	Tissue	55	44	median	16	Surgery	0.529	0.272	1.031	9
Chen DS	2018	China	Asian	LC	hsa_circ_100395	/	D	Tissue	35	34	mean	150	Surgery	0.61	0.25	1.49	7

NSCLC, nonsmall cell lung cancer; LAC, lung adenocarcinoma; LC, lung cancer; U, upregulated expression; D, downregulated expression; OS, overall survival; HR, hazard ratio; LL, lower limit; UL, upper limit; NOS, Newcastle-Ottawa Scale.

**Table 3:** The results of the diagnostic analysis.

First author	Sensitivity	Specificity	LR+ (95%CI)	LR- (95%CI)	DOR	AUC
Li JP	0.76 (0.66-0.84)	0.72 (0.68-0.84)	2.69 (1.91-3.80)	0.33 (0.23-0.49)	8.08 (4.18-15.63)	
Zhang YN	0.80 (0.65-0.90)	0.73 (0.58-0.85)	3.00 (1.81-4.98)	0.27 (0.15-0.50)	11.00 (4.11-29.45)	
Zhang SY	0.87 (0.74-0.95)	0.85 (0.71-0.94)	5.71 (2.86-11.41)	0.15 (0.07-0.33)	37.14 (11.46-120.42)	
Zhu XL	0.76 (0.61-0.87)	0.80 (0.66-0.90)	3.70 (2.08-6.58)	0.31 (0.18-0.51)	12.02 (4.64-31.16)	
Zong L	0.81( 0.68-0.90)	0.89 (0.78-0.96)	7.67 (3.56-16.52)	0.22 (0.13-0.37)	35.55 (12.17-103.79)	
Pooled	0.80 (0.74-0.84)	0.80 (0.73-0.86)	3.97 (2.80-5.62)	0.26 (0.19-0.34)	15.51 (8.76-27.47)	0.85 (0.82-0.88)
*I* ^*2*^	0	54.09%	17.25%	12.03%	93.33%	

LR+: positive likelihood ratios; LR–, negative likelihood ratios; DOR, diagnostic odds ratios; AUC, area under curve; I^2^, inconsistency index.

**Table 4:** The results of the prognostic subgroup analysis.

Subgroup	No. of studies	HR	LL	UL	P	I^2^	P for heterogeneity
Total	1544	1.70	1.26	2.29	0.001	72.8%	< 0.001
Upregulated	1323	2.17	1.74	2.72	< 0.001	43.2%	0.034
Downregulated	221	0.52	0.34	0.80	0.002	0	< 0.001
Analysis methods							
Multivariate analysis	980	1.79	1.17	2.73	0.007	84.8%	< 0.001
Univariate analysis	564	1.56	1.13	2.16	0.007	0	0.510
Follow-up time							
≥ 5 years	1280	1.76	1.28	2.43	0.001	72.5%	< 0.001
< 5 years	258	1.52	0.67	3.47	0.319	74.9%	0.009

HR, hazard ratio; LL, lower limit; UL, upper limit.

## Data Availability

The data used to support the findings of this study are included within the article.

## Abbreviations

AUC: Area under the curve
CDR1as: Cerebellar degeneration-related protein 1 transcript
CI: Confidence interval
D: Downregulated expression
DOR: Diagnostic odds ratio
FN: False negative
FP: False positive
HR: Hazard ratio
LAC: Lung adenocarcinoma
NLR: Negative likelihood ratio
NSCLC: Nonsmall cell lung cancer
U: Upregulated expression
OS: Overall survival
PLR: Positive likelihood ratio
QUADAS: Quality Assessment of Diagnostic Accuracy Studies
qRT-PCR: Real-time polymerase chain reaction
SROC: Summary receiver operating curve
TN: True negative
TP: True positive.

## Supplementary Materials

Supplementary Materials Figure S1: quality evaluation of diagnostic accuracy for the enrolled studies as well as risk of bias and applicability concerns' (A) graph and (B) summary.
