# Supplementary material for: Potential Diagnostic and Prognostic Biomarkers of Circular RNAs for Lung Cancer in China
Source: Biomed Res Int. 2019 Aug 25;2019:8023541. doi: 10.1155/2019/8023541 (PMC6732606; doi:10.1155/2019/8023541)
Supplement: Supplementary Materials — Figure S1: quality evaluation of diagnostic accuracy for the enrolled studies as well as risk of bias and applicability concerns' (A) graph and (B) summary. [file 8023541.f1.pptx]

## Slide 1
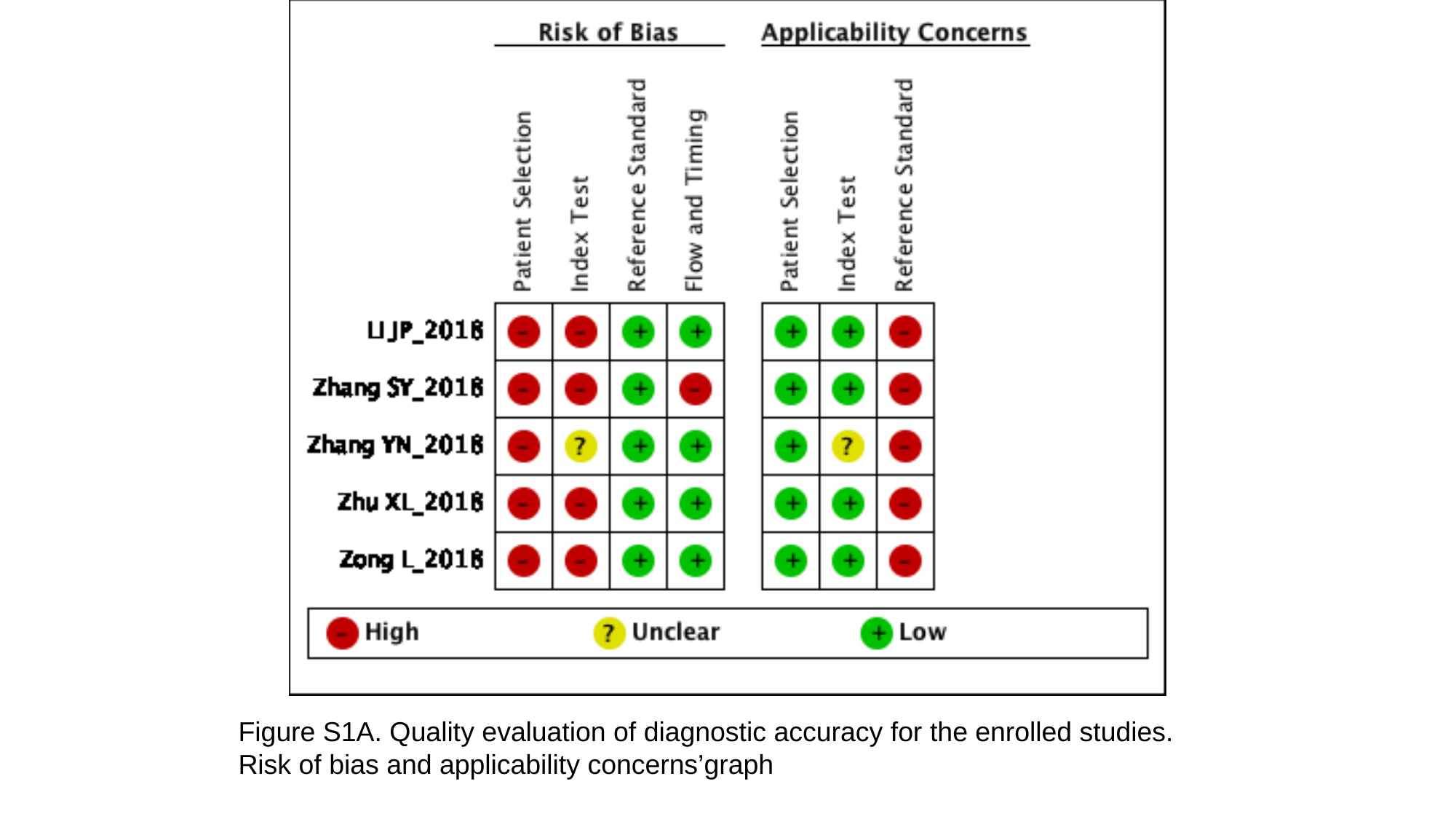

Figure S1A. Quality evaluation of diagnostic accuracy for the enrolled studies. Risk of bias and applicability concerns’graph

## Slide 2
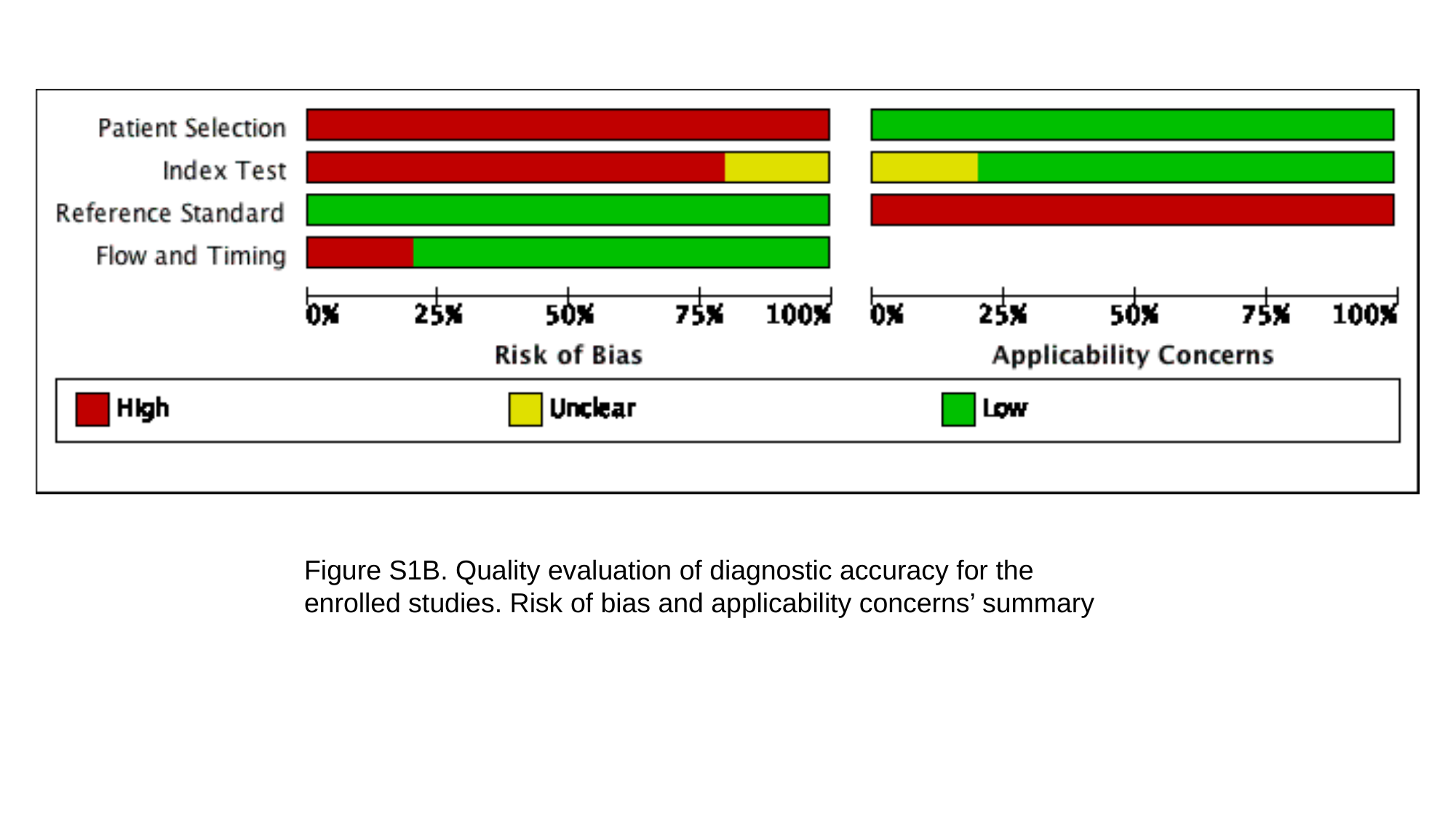

Figure S1B. Quality evaluation of diagnostic accuracy for the enrolled studies. Risk of bias and applicability concerns’ summary
